# The Diagnostic Value of Global Longitudinal Strain (GLS) on Myocardial Infarction Size by Echocardiography: A Systematic Review and Meta-analysis

**DOI:** 10.1038/s41598-017-09096-2

**Published:** 2017-08-30

**Authors:** Kai-yue Diao, Zhi-gang Yang, Min Ma, Yong He, Qin Zhao, Xi Liu, Yue Gao, Lin-jun Xie, Ying-kun Guo

**Affiliations:** 1 Department of Radiology, State Key Laboratory of Biotherapy, West China Hospital, Sichuan University, Chengdu, China; 2 Department of Cardiology, West China Hospital, Sichuan University, Chengdu, China; 3 Department of Cardiology, The Sixth People’s Hospital of Chengdu, Chengdu, China; 4 Department of Radiology, Key Laboratory of Birth Defects and Related Diseases of Women and Children of Ministry of Education, West China Second University Hospital, Sichuan University, Chengdu, China

## Abstract

A systematic review and meta-analysis of prospective randomized studies were performed to evaluate the diagnostic value of measuring global longitudinal strain (GLS) using speckle tracking echocardiography (STE) in determining myocardial infarction (MI) size, which is usually measured based on late gadolinium enhancement (LGE) by cardiovascular magnetic resonance (CMR). Eleven trials with a total of 765 patients were included. The pooled correlation was 0.70 (95% CI: 0.64, 0.74) between two-dimensional (2D) GLS and the LGE percentage, and it was 0.55 (95% CI: 0.19, 0.78) for three-dimensional (3D) GLS. Pooled diagnostic estimates for 2D GLS to differentiate an MI size >12% were as follows: sensitivity, 0.77 (95% CI: 0.61, 0.90); specificity, 0.86 (95% CI: 0.68, 0.96); positive likelihood ratio (PLR), 8.13 (95% CI: 1.90, 26.61); negative likelihood ratio (NLR), 0.28 (95% CI: 0.10, 0.54); and diagnostic odds ratio (DOR), 39.87 (95% CI: 4.12, 172.83). The estimated area under the curve (AUC) of the summary receiver operating characteristic (SROC) curve was 0.702. The 2D STE results positively correlated with the infarction size quantified by CMR for patients who had experienced their first MI. This approach can serve as a good diagnostic index for assessing infarction area. However, more consolidated STE studies are still needed to determine the value of 3D STE.

## Introduction

Myocardial infarction (MI), and especially acute MI (AMI), is a major life-threatening cardiovascular event that places huge disease and economic burdens on society^1^. Assessment of the size and distribution of the infarction area after revascularization therapy can facilitate prompt and appropriate clinical intervention. Biomarkers such as troponin and creatine kinase are mainly used for AMI identification but lack myocardial specificity and may overestimate the infarction size (IS)^2^. Conventional bedside echocardiography provides a quick and general overview of the state of the myocardium, but common indexes such as the left ventricle ejection fraction (LVEF) fail to detect minimal and early pathological changes^3^. Late gadolinium enhancement (LGE) in cardiovascular magnetic resonance (CMR) provides quantitative details for myocardial fibrosis and serves as a promising tool for infarction area evaluation^4, 5^. However, widespread application of CMR remains limited due to its limited availability in poor areas, long scanning time and special requirements for breath-holding during an examination^6^.

Myocardial strain is a quantitative index based on measuring myocardial deformation during a cardiac cycle^7^. Major tools for detecting changes in myocardial strain include CMR tagging, CMR feature tracking (FT-CMR)^6^ and speckle tracking echocardiography (STE)^8^. Previous studies have shown an advantage of strain in sensitively and accurately diagnosing and assessing IS compared to traditional functional indexes^9^. However, the degree to which strain analysis can reflect the infarction areas quantified by CMR as well as the diagnostic accuracy of this analysis is still under dispute. In the past 3 years in particular, newly developed three-dimensional (3D) STE has overcome the inherent shortcomings of two-dimensional (2D) STE but has shown uneven diagnostic performance in different studies. Until now, no systematic reviews or meta-analyses have been conducted to clarify and address these issues. Thus, we conducted a meta-analysis and systematic review of the studies evaluating the performance of STE in detecting and assessing infarctions after AMI.

## Results

### Search strategy and study selection

**Figure 1:**
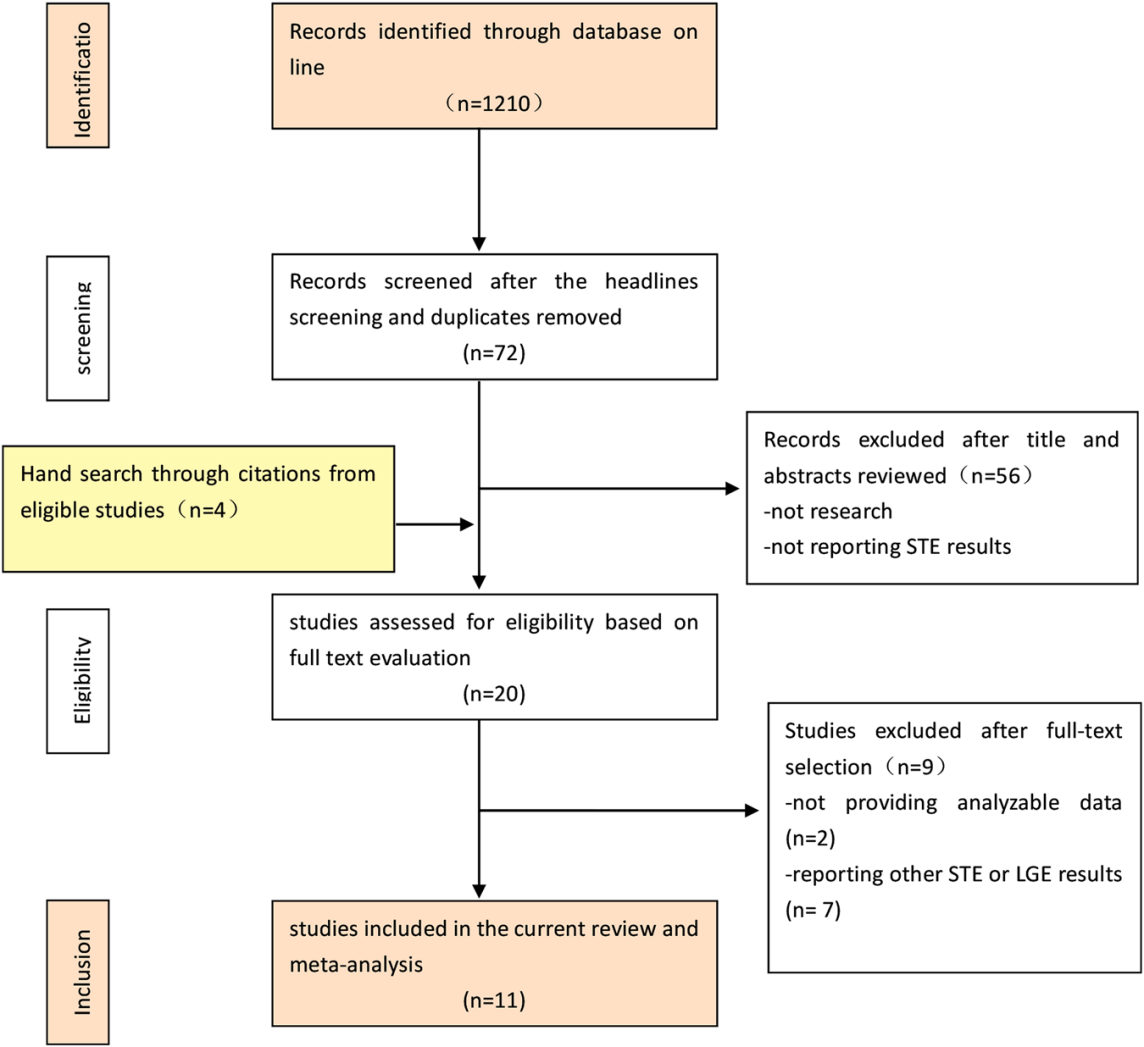
Flow diagram of literature search.

The online search initially yielded 1210 literature citations. Of those citations, 1138 were excluded after reviewing the titles and keywords because of non-relevance or repetition. Two authors (M.M. and K.D.) reviewed 72 abstracts and selected 16 studies for full-text evaluation. Simultaneously, another author (X.L.) reviewed the citations in the 16 studies and added 4 more papers for full-text evaluation. Further reading excluded papers according to the previously defined criteria. Seven studies were excluded due to not using the required index for interpreting STE and LGE results, and 2 studies were excluded for a lack of data needed for the meta-analysis. The Preferred Reporting Items for Systematic Reviews and Meta-Analyses (PRISMA) flow chart depicting the process for the systematic literature search and study selection is shown in Fig. 1.

### Study characteristics

Ultimately, 11 prospective studies published between 2007 and 2016 were chosen for systematic review. Among these studies, 10 quantitatively estimated the correlation between the strain results analysed by echocardiography and the scar size measured on CMR^10–19^, and 8 reported the overall diagnostic value of STE in determining IS^20^. All the studies used 1.5 T MRI and performed LGE following reference standards (0.1 to 0.2 mmol/kg gadolinium; 10 to 20 min for imaging) for IS quantification (Table 1).

**Table 1:** Included Studies and the Basic Characteristics.

Study	Year	Journal	Study design	N	Population	Echo	CMR
T. V., *et al*.	2007	JACC	single center, prospective	30	1st-time STEMI	2D, EchoPAC	10–20 mins; 0.1 mmol/kg; 1.5 T
C. E., *et al*.	2010	CircCardiovasc Imaging	single center, prospective	61	1st-time NSTEMI	2D; EchoPac	10–20 mins; 0.1–0.2 mmol/kg; 1.5 T
N. M., *et al*.	2011	European Journal of Echocardiography	single center, prospective	163	1st-time STEMI	2D; EchoPAC	15 mins; 0.15 mmol/kg; 1.5 T
D. H., *et al*.	2012	The American Journal of Cardiology	single center, prospective	25	MI with LVEF < 50%	2D; 3D Artida4D	10–20 mins; 0.2 mmol/kg; 1.5 T
A. T., *et al*.	2013	Echocardiography	single center, prospective	58	1st-time MI	2D; 3D EchoPAC	10–20 mins; 0.15 mmol/kg; 1.5 T
W. Z., *et al*.	2013	Echocardiography	single center, prospective	26	1st-time STEMI	3D; Artida	N/A; 1.5 T
C. S., *et al*.	2013	European Heart Journal	single center, prospective	20	1st-time STEMI with LVEF > 40%	2D; 2D CPA	15 mins; 0.1 mmol/kg; 1.5 T
M. G., *et al*.	2013	Coronary Artery Disease	single center, prospective	39	1st-time anterior STEMI	2D; EchoPAC	NA; 0.1 mmol/kg; 1.5 T
L. B., *et al*.	2014	PlosOne	single center, prospective	41	1st-time STEMI	2D; EchoPAC	10–12 mins; 0.2 mmol/kg; 1.5 T
M. L., *et al*.	2015	Clinical Medicine Insights: Cardiology	multi-center, prospective	30	1st-time NSTEMI	2D; Q lab	10-20mins; 0.15mmol/kg;1.5T
M. F. A., *et al*.	2016	Neth Heart J	single center, prospective	80	1st MI with LVEF<50%	3D; Artida4D	10-15mins; 0.2 mmol/kg;1.5T

STEMI: ST-elevation: ST-segment elevation myocardial infarction; LVEF: left ventricular ejection fraction; MI: myocardial infarction.

In total, 765 patients (with a mean age of 58.8 years) were included; more detailed baseline characteristics are presented in Table 2. LVEF, hypertension (HTN), diabetes mellitus (DM) and current smoking status were the most concerning and commonly recorded items.

**Table 2:** Patient Characteristics.

Study	Age	Men, %	HTN	Diabetes	Smokers	Anterior/Inferior infarction	LVEF _echo	LVEF_CMR
T. V., *et al*.	57.7 ± 8.6	23(76.7%)	N/A	N/A	N/A	N/A	42 ± 9	NA
C. E, *et al*.	57.7 ± 8.6	48(78.7%)	24(39.3%)	7(11.5%)	27(44.3%)	15(24.6%)(LAD)/37(60.7%)(LCX + RCA)	55.6 ± 8.0	NA
N. M., *et al*.	60	148(82%)	50(28%)	10(6%)	140(77%)	79(44%)/84(56%)	55(49–62)	57(50–63)
D. H., *et al*.	62 ± 16	20(80%)	11(44%)	4(16%)	13(52%)	17(68%)/8(32%)	NA	41 ± 9
A. T., *et al*.	55 ± 13	44 (75.9%)	10(17.2%)	6(10.3%)	29(50%)	23(39.7%)(LAD)/35(60.3%)(LCX + RCA)	55.2 ± 6.4	NA
W. Z., *et al*.	56.3 ± 11.1	15(57.7%)	N/A	N/A	N/A	26(100%)	47.1 ± 6.8	NA
C. S., *et al*.	57 ± 10	18(90%)	10(53%)	1(5.9%)	12(59%)	N/A	48 ± 4.5	50 + 10
M. G., *et al*.	59 ± 10	29(74%)	33(85%)	5(13%)	23(59%)	39(100%)/0	47 ± 9	NA
L. B., *et al*.	57 ± 12	34(82%)	16(39%)	8(19%)	23(56%)	27(66%)(LAD)/14(34%)(LCX + RCA)	51.2 ± 7.3	50
M. L., *et al*.	52.7 ± 9.7	28(93.3%)	7(23.3%)	6(20%)	26(86.7%)	N/A	56.9 ± 5.5	NA
M. F. A., *et al*.	63 ± 12	65(79%)	42(53%)	20(25%)	N/A	N/A	39 ± 10	40 ± 8

HTN: hypertension; LVEF: left ventricular ejection fraction; CMR: cardiovascular magnetic resonance; NA: not available; LAD: left anterior descending branch; LCX: left circumflex branch; RCA: right coronary artery.

**Figure 2:**
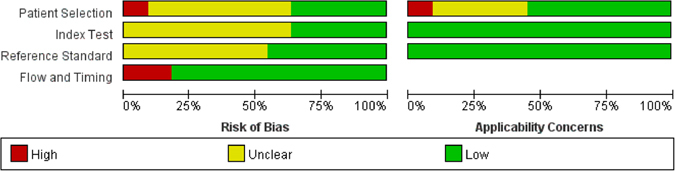
Study quality evaluated by QUADAS-2 tool. Grouped bar chart displays the cumulative score of the 11 included studies for each field of the QUADAS questions. Green bar = “low” risk, yellow bar = “unclear” risk, and red bar = “high” risk.

The quality assessment showed an acceptable overall risk of bias and applicability concerns. All studies provided detailed inclusion and exclusion criteria, except for the study by M.F.A. *et al*. Three studies failed to use credible time intervals between echocardiography and CMR tests. Only three studies confirmed that both the echocardiography and the CMR tests were conducted in a blinded manner. Further details are provided in Fig. 2.

### Correlation between STE and scar size

**Figure 3:**
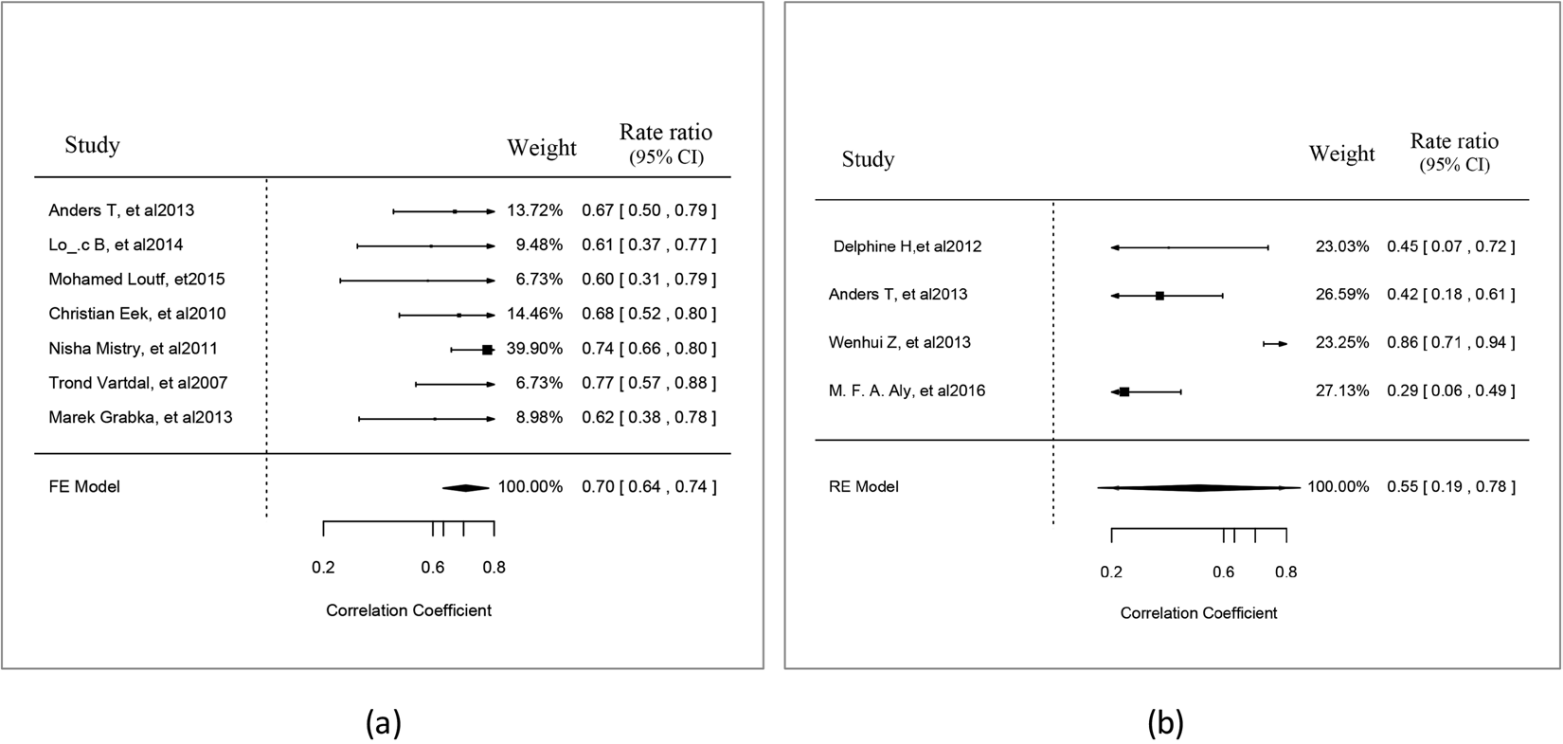
Forest plot of the correlation coefficients between the GLS on 2D/3D STE and the MI area on CMR.

CIs were computed for all of the selected 11 studies (Table 3). Among these studies, 7 reported 2D STE results, whereas 4 studies reported 3D STE results. GLS was the only shared reported item and was the most correlated item according to the results of the selected studies. The pooled r-value for the 7 studies for reporting correlations between 2D GLS assessed by echocardiography and the LGE area measured on CMR was 0.70 (95% CI: 0.64, 0.74), without notable heterogeneity (chi-squared = 4.45, P = 0.62 for the Q test and I^2 = 1.44%) (Fig. 3a). However, the r-value for the 4 studies that applied 3D STE was 0.55 (95% CI: 0.19, 0.78), and there was notable heterogeneity (chi-squared = 17.38, P = 0.0006 for the Q test and I^2 = 85.99%) (Fig. 3b). Leave-one-out diagnostics were performed to test the source of heterogeneity, and a study by Wenhui Z *et al*. showed a significant influence, excluding a pooled r-value of 0.38 (95% CI: 0.22, 0.54) that could be reached without heterogeneity (chi-squared = 0.9425, P = 0.6242 for the Q test and I^2 = 0.00%).

**Table 3:** The Individual Correlation Coefficient from Each Study and the Calculated 95% Confidential Interval (CI).

Study	Number of patients	Time_interval	Correlation coefficient	
			|r|	CI
2D GLS				
TV,2007	30	<1 day	0.77	[0.57, 0.88]
CE,2010	61	>1 month	0.68	[0.52, 0.80]
NM, 2011	163	<1 day	0.74	[0.66,0.80]
AT,2013	58	<1 day	0.67	[0.50, 0.79]
MG, 2013	39	>1 month	0.62	[0.38,0.78]
LB, 2014	41	>1 month	0.61	[0.37,0.77]
ML, 2015	30	<5 day	0.60	[0.31,0.79]
3D GLS				
DH,2012	25	<1 day	0.45	[0.07,0.72]
AT, 2013	58	<1 day	0.42	[0.18,0.61]
WZ, 2013	26	<1 day	0.86	[0.71, 0.94]
MFA,2016	71	<1 day	0.29	[0.06, 0.49]

A funnel plot was performed to test for publication bias, and no prominent bias was found. Considering that the number of selected studies was less than 10 and that no reliable test is available at this level, the trim-and-fill method was performed to test the results after considering possible publication bias. The corrected r-values for 2D GLS and 3D GLS were 0.88 (95% CI: 0.80, 0.98) and 0.61 (95% CI: 0.19, 1.04), respectively.

### Diagnostic accuracy of STE

The data extracted from the selected studies are shown in Table 4.

**Table 4:** The Extracted Data from the Studies Reporting Diagnostic Indexes for Using 2D GLS.

Study	Cut-off	TP	FP	TN	FN	AUC	Sensitivity	**Specificity**
0%								
C. S., 2013	−12	49	42	148	11	0.673	82%	78%
12%								
C. E., 2010	−13.8	11	2	46	2	0.95(0.86–0.99)	85%	96%
A. T., 2013	−16.5	17	7	28	8	0.80 (0.68–0.92)	68%	85%
M. K., 2015	−11.3	10	4	14	2	0.82 (0.67–0.98)	77.8%	83.3%
15.7%								
N. M., 2011	−15.9	22	11	120	10	0.81(0.72–0.91)	68.8%	91.5%
20%								
M. G., 2013	−12.3	18	2	15	4	0.83	82%	87%
50%								
A. T., 2013	−14.4	59	47	266	15	0.88(0.83–0.93)	80%	85%
C. S., 2013	−11	29	45	165	10	0.66	75%	78%
M. L., 2015	−9.1	31	86	225	12	NA	71.8%	72.4%

**Figure 4:**
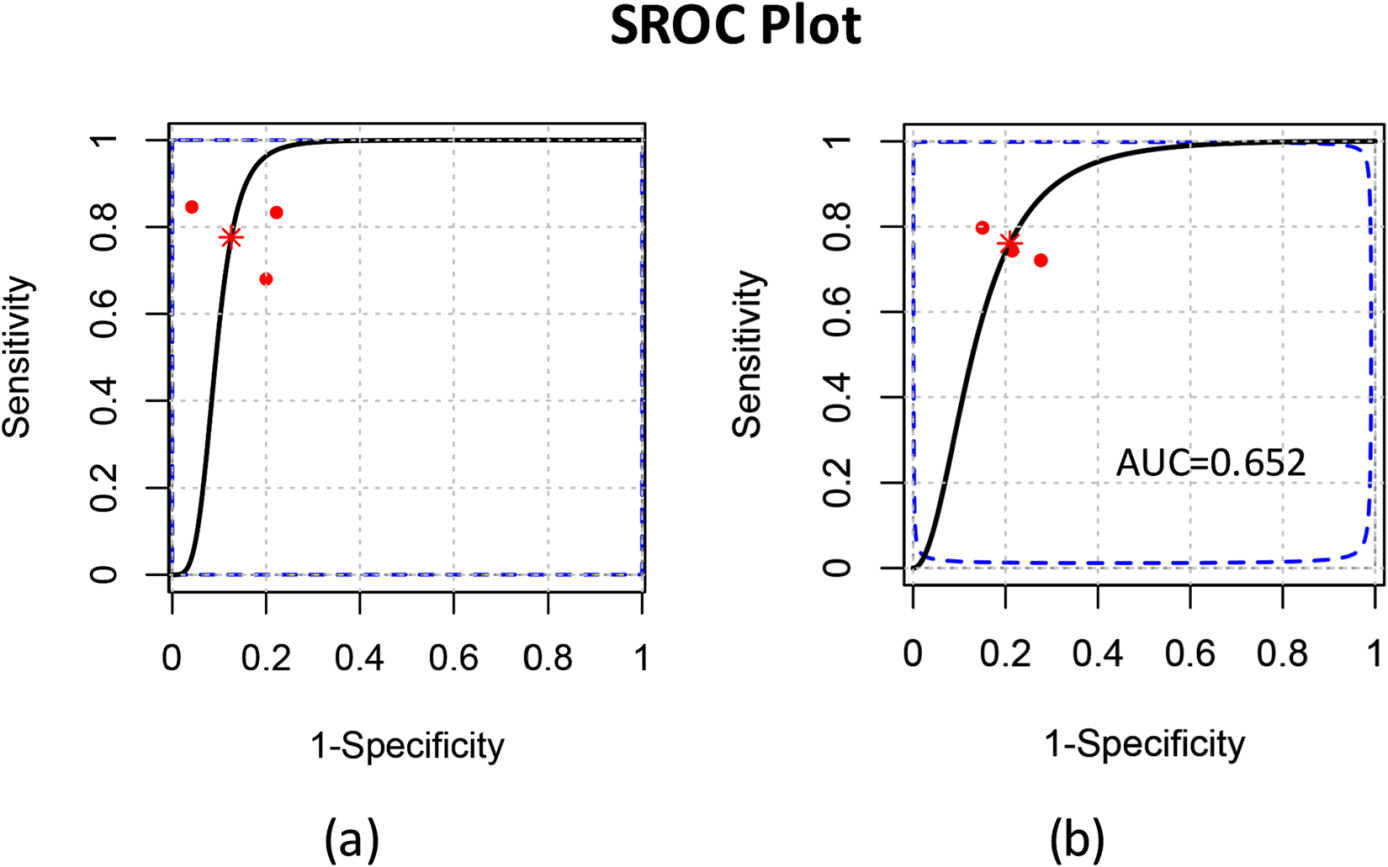
Summary receiver operator characteristic (SROC) of 2D GLS on differentiating MI area: (**a**) under or more than 12% shows an AUC (area under the curve) of 0.702; (**b**) under or more than 50% shows an AUC (area under the curve) of 0.652.

For differentiating maximal (>12%) and minimal (<12%) ISs, the selected studies showed sensitivities ranging from 0.68 to 0.85. The specificities ranged from 0.83 to 0.96, and the area under the curve (AUC) values ranged from 0.80 to 0.95. Pooled estimates were as follows: sensitivity, 0.77 (95% CI: 0.61, 0.90); specificity, 0.86 (95% CI: 0.68, 0.96); positive likelihood ratio (PLR), 8.13 (95% CI: 1.90, 26.61); negative likelihood ratio (NLR), 0.28 (95% CI: 0.10, 0.54); and diagnostic odds ratio (DOR), 39.87 (95% CI: 4.12, 172.83). The summary receiver operating characteristic (SROC) curve summarized the overall diagnostic accuracy, showing a trade-off between sensitivity and specificity in which the calculated AUC was 0.702 (Fig. 4a).

For assessing the transmurality of the infarction area, the selected studies showed sensitivities ranging from 0.72 to 0.80 and specificities ranging from 0.72 to 0.85, and the AUC values ranged from 0.66 to 0.88. The pooled estimates for 2D GLS were as follows: sensitivity, 0.76 (95% CI: 0.66, 0.84); specificity, 0.79 (95% CI: 0.69, 0.87); PLR, 3.79 (95% CI: 2.19, 6.52); NLR, 0.31 (95% CI: 0.19, 0.47); and DOR, 13.46 (95% CI: 4.94, 30.37). The corresponding SROC curves are shown in Fig. 4b, and the estimated AUC was 0.652.

## Discussion

MI size has long played a major role in clinical decision-making. In this context, accurate and quick morphological and functional evaluations are profoundly significant. Our study, which included a meta-analysis and systematic review, is the first to summarize the previous research on the diagnostic value of GLS determined by performing 2D and 3D STE in MI patients.

First, our results showed good overall correlation between the 2D GLS and the LGE results, which are superior to conventional indexes, including both troponin level^21, 22^ and LVEF^23^. Second, for using GLS results to differentiate large and small infarctions or to predict transmural MI, the pooled diagnostic value was inferior to the results for each individual centre. The pooled DOR, PLR and NLR results showed a better diagnostic performance for differentiating a 12% MI size than for differentiating a 50% MI size. The DOR results showed that GLS had a moderately accurate diagnostic value for differentiating ISs >12% and <12%. For differentiating transmural infarctions, however, although the DOR was >1, the results still indicated a relatively poor performance. The cut-off values for GLS are still variable and might depend on the experience of clinicians at different centres. Additionally, previous studies on both GLS and other diagnostic indexes did not show better results for diagnostic methods other than GLS^11^. Additionally, in assessing the viability of the myocardium after MI, LVEF might have better performance^17^.

Classical myocardial theory was used to demonstrate a three-layer model, including inner and outer oblique fibres and a more horizontal middle layer. The pumping function of the heart has been attributed to the contractions of fibres in different orientations. Pathological changes in MI usually start from the endocardium and develop to the outmost layer with prolongation of the ischaemia time. According to a previous study^7^, the change in longitudinal strain is most prominent during the pre-ejection and ejection phases. Therefore, even the circumferential strain (CS) of the myocardium at the endocardial layer is twice that of the myocardium at the epicardial layer^24^, and most studies still show better sensitivity to dysfunctional change for GLS than for global CS. A reason might be that the ultrasound beam of the probe is longitudinal and thus parallel to the GLS, which helps to achieve better resolution in the longitudinal direction, whereas GCS is calculated from the short axis and is thus inevitably affected by low resolution in the basal and apex layers. Advancement in algorithms and methodologies might improve the accuracy of GCS in the future.

According to our results, 2D GLS has better performance than 3D GLS as well as when compared to results generated by CMR. Interestingly, for the studies using both 2D and 3D STE, better performance was seen for 3D GCS calculations than for 2D GCS^16, 19^. The two technologies have two primary differences that might explain this result. First, compared to 2D STE, real-time 3D STE overcomes the cross-lane tracking shortcoming of 2D STE to allow for a quick overall evaluation of the heart. However, this feature is limited by the sole capacity for a full-wall thickness assessment, in contrast to the multi-layered assessment provided by 2D STE. Second, strain change in the longitudinal direction can be mainly attributed to the oblique layers of sub-endocardium and can lead to a preference for single-layer analysis, although transverse strain varies in all the layers of myocardium, which might be more suited to more thorough data collection throughout the myocardium. Thus, 3D STE might be more suitable than 2D STE for GCS evaluation and may not be accurate enough for GLS evaluation. However, 3D STE is still a promising developing new technology, and the heterogeneity among previous 3D STE studies demonstrates that more studies are still needed to clarify the differences between 2D and 3D STE as well as the technical advancements in image analysis for 3D STE.

Other than 3D STE, another promising strain analysis tool is FT-CMR. Compared to the traditional CMR tagging technique, FT-CMR borrows the 2D deformation analysis algorithm from 2D STE and adapts it to a CMR cine steady-state free precession (SSFP) sequence, which is exempt from additional scans or contrast injection. However, FT-CMR tracks deformation more on the endocardial border, whereas STE is concentrated on intra-myocardial changes. A previous review also reported that the strain analysed by FT-CMR was lower than that analysed by STE^25^. Additionally, software for FT-CMR is relatively rare, leading to highly identical parameters recorded among studies, whereas the software and assessment standards for STE are highly variable^26^. Thus, arbitrarily deciding which technique is better is not appropriate, and more studies are still needed. Furthermore, the development of a new strain calculation was also noted during the present review.

### Limitations

For this meta-analysis, there are two main limitations. First, a relatively small group of studies was included for assessment of diagnostic accuracy. Due to the different thresholds used in different studies, we failed to achieve a pooled result based on more studies. Second, heterogeneity was observed for 3D STE studies. As discussed above, the standard for applying STE analysis is deficient, and its revision is a goal of the new European Society of Cardiovascular Imaging and the American Society of Echocardiography strain standardization task force. More studies with a relatively standard procedure are needed to achieve more credible results.

In conclusion, GLS measured by 2D STE had a good correlation with the IS quantified by CMR for patients with first-time MI and can serve as a clinical diagnostic factor for assessing the MI area. Meanwhile, the 3D STE approach showed inferior diagnostic value compared to 2D STE, and more consolidated 3D STE studies are still needed to clarify the value of this technology.

## Methods

Following PRISMA standards^27^, our main process of review and assessment was conducted as described below.

### Search strategy

Two independent reviewers (L.X. and M.M.) separately searched PubMed, Embase, and the Cochrane Central Register for studies using the following keywords: “myocardial ischemia” OR “coronary artery disease” OR “myocardial infarction” AND “speckle tracking echocardiography” OR “two-dimensional echocardiography” OR “three-dimensional echocardiography” AND “strain” AND “size” OR “area” OR “viable myocardium” AND “evaluation” OR “assessment” OR “diagnosis”. We confined the publication date range to 2005 until present. We also verified and manually searched for certain papers in the reference lists of the selected studies. We put no limits on language.

### Study selection

Studies were included if they 1) randomly and prospectively enrolled patients with one MI, 2) performed standard methods to acquire LGE as the reference for infarction area quantification, 3) performed either 2D or 3D STE or both and recorded GLS for myocardial function assessment, and 4) performed credible statistical methods to study the correlation between STE and CMR or to evaluate the diagnostic value of GLS determined by STE or both.

Two main types of studies were considered in the literature search. The first type performed a scar size assessment in which the correlations between the results from echocardiography and CMR were studied. The other type reported the diagnostic value of echocardiography in differentiating large and small MIs or recognizing transmural MI.

To define the borderline size of the scar tissue, the percentage of the LGE area in the left ventricle was taken as the standard interpretation. A value of 50% was mainly used to define transmural MI, considering that previous studies noted this value as the cut-off for differentiating myocardial recovery ability after revascularization^4^. In addition to that value, 12% was the preferred threshold for differentiating maximal and minimal MIs because it has been shown to have a notable correlation with mortality^28^.

### Quality assessment

Patient selection, the index and reference tests, and flow and timing were mainly considered to assess study quality while referring to the items in the Quality Assessment of Diagnostic Accuracy Studies (QUADAS-2)^29^.

### Data extraction

Two other reviewers (Q.Z. and Y.G.) carefully reviewed the full text and extracted the intended data from the selected studies. Any discrepancies between the two were referred to another reviewer (K.D.) for a final decision. We recorded the patients’ basic information, the target correlation factor and the 2 × 2 data for reporting diagnostic accuracy as well as the different borderline LGE results or STE cut-off values used. Furthermore, we recorded the methodological techniques mentioned by the authors in each study and summarized these techniques in our results.

For studies that did not provide 2 × 2 data, we used the summary results to calculate the responding true-positive, true-negative, false-positive and false-negative values.

### Data analysis

The baseline variables for the patients are given as the proportion (percentage), and continuous variables are given as the mean (SD) or median (range). The time intervals for the different measurement methods were calculated and are reported in the format of time ranges.

For studies reporting correlation factors between the scar sizes measured by STE and LGE, we calculated the CI for each correlation coefficient and the pooled r-value using a method described in a previous study^30^.

For studies reporting the diagnostic accuracy of STE, we computed the pooled sensitivity, specificity, PLR, NLR, and DOR and generated a forest plot accordingly. The DOR was considered the major diagnostic accuracy assessment index independent of disease prevalence, with a DOR > 25 considered to be moderately accurate and a DOR > 100 considered to be highly accurate^31^. An SROC curve was calculated with the sensitivity and specificity values provided by every single study.

Statistical heterogeneity was calculated as I^2, and if the heterogeneity was significant, the results of the random-effect model were reported. We also performed a sensitivity test to see if excluding any study provided lower heterogeneity. A funnel plot was used to measure publication bias, and Egger’s^32^ test was used as well if the tested groups included more than 10 studies. The trim-and-fill method was used if prominent bias was observed. The meta-analyses were performed using R Project (3.3.1) with the freeware package (meta4diag and metafor, 2016).
